# Supplementary material for: Computer prediction and genetic analysis identifies retinoic acid modulation as a driver of conserved longevity pathways in genetically diverse Caenorhabditis nematodes
Source: eLife. 2025 Dec 23;13:RP104375. doi: 10.7554/eLife.104375 (PMC12726832; doi:10.7554/eLife.104375)
Supplement: Supplementary file 1. — Tables containing (a) sources of experimental variation across trials, genes significantly (b) up- and (c) downregulated by atRA treatment, and (d) WaRG comparisons across hsf-1 and aak-2 gene expression analyses. [file elife-104375-supp1.docx]

Supplemental File 1a—Pooled sources of variance for manual atRA lifespan replicated across the three CITP tested sites.

Reproducibility of manual lifespan assays within and among labs of the CITP. Variance estimates were estimated using a general linear model. Bolded sources of variance categories sum the component numbers presented immediately beneath. Individual variation represents variability unassignable to a specific source of variance.

| **Source of Variation** | |
| --- | --- |
| Genetic Variation | 22.8 |
| Among species | 17.3 |
| Among strains w/in species | 3.9 |
| Species x Compound | 1.6 |
| Strain x Compound | 0 |
| Reproducibility Among Labs | 3.9 |
| Lab | 0 |
| Lab x Species | 0 |
| Lab x Strain | 3.6 |
| Lab x Compound | 0.3 |
| Reproducibility Within Labs | 20 |
| Among trials | 5.8 |
| Among experimenters w/in trials | 7.1 |
| Among plates w/in experimenters | 7.1 |
| Individual Variation | 53.2 |
| Total | 100 |

Supplemental File 1b— Genes upregulated with a LFC >3 in response to atRA in wildtype CITP N2

|  | | | | **Putative target of known regulatory pathway** | |
| --- | --- | --- | --- | --- | --- |
| **Gene** | **LFC** | **P-value** | **FDR** | **IIS (PI3K/Akt)** | **Nrf2 (p38 MAPK)** |
| ***E02C12.10*** | 9.9 | 4.62E-18 | 5.89E-14 |  |  |
| *C52A10.2* *(cest-35.2)* | 8.1 | 2.5E-16 | 7.35E-13 | *daf-2* (Gao et al., 2018; Senchuk et al., 2018; Sonoda et al., 2016), *daf-16* (Hibshman et al., 2017; Kaplan et al., 2015) | *pmk-1* (Bond et al., 2014) |
| *irld-14* | 7.4 | 2.8E-06 | 0.000111 | *daf-2* (Gao et al., 2018) |  |
| *gpa-6* | 6.5 | 2.68E-14 | 4.27E-11 |  |  |
| *gba-2* | 6.1 | 2.88E-16 | 7.35E-13 | *daf-2* (Knutson et al., 2016; M. Seo et al., 2015) | *skn-1* (Nhan et al., 2019), *pmk-1* (Fletcher et al., 2019) |
| *R05D8.9* | 5.8 | 1.05E-11 | 7.06E-09 | *daf-16* (Kaplan et al., 2015; Li et al., 2019) | *skn-1*(Nhan et al., 2019) |
| *cyp-33C2* | 5.7 | 4.37E-07 | 2.67E-05 | *daf-2* (Chen et al., 2013; Knutson et al., 2016; Sonoda et al., 2016), *daf-16* (Chen et al., 2013) |  |
| *cyp-35A4* | 5.7 | 7.74E-17 | 3.29E-13 | *daf-2* (Knutson et al., 2016) | *pmk-1* (Bond et al., 2014; Fletcher et al., 2019) |
| *T16G1.6* | 5.1 | 1.1E-14 | 2.01E-11 | *daf-2* (Gao et al., 2018; M. Seo et al., 2015; Son et al., 2018), *daf-16* (Delaney et al., 2017; Hibshman et al., 2017; Kaplan et al., 2015) | *pmk-1* (Fletcher et al., 2019) |
| ***E02C12.12*** | 5.1 | 1.44E-06 | 6.69E-05 |  |  |
| *C40H1.8* | 4.7 | 2E-12 | 1.96E-09 | *daf-2* (Senchuk et al., 2018), *daf-16* (Li et al., 2019) |  |
| *fil-1* | 4.4 | 1.23E-12 | 1.31E-09 | *daf-2* (M. Seo et al., 2015), *daf-16* (Kaplan et al., 2015; McCormick et al., 2012; Wu et al., 2016) | *skn-1* (Dodd et al., 2018; Kaushik et al., 2021; Nhan et al., 2019; Steinbaugh et al., 2015; Wu et al., 2016), *pmk-1* (Fletcher et al., 2019) |
| ***E02C12.6*** | 4.3 | 1.57E-10 | 6.27E-08 |  | *skn-1* (Kaushik et al., 2021; Steinbaugh et al., 2015) |
| *F15H10.7* | 4.3 | 3.68E-08 | 4.36E-06 | *daf-2* (Kaletsky et al., 2016) | *skn-1* (Kaushik et al., 2021), *sek-1* (Wu et al., 2019) |
| *hrg-7* | 3.6 | 5.84E-17 | 3.29E-13 | *daf-2* (Knutson et al., 2016; M. Seo et al., 2015; Son et al., 2018), *daf-16* (Delaney et al., 2017; Kaplan et al., 2015; S.-J. Lee et al., 2009; Wu et al., 2016) | *skn-1* (Kaushik et al., 2021) |
| *cyp-13A7* | 3.5 | 4.44E-08 | 4.83E-06 | *daf-2* (Gao et al., 2018), *daf-16* (Delaney et al., 2017) | *skn-1* (Kaushik et al., 2021) |
| *clec-227* | 3.4 | 1.85E-08 | 2.66E-06 | *daf-2* (Senchuk et al., 2018), *daf-16* (Kaplan et al., 2015; Kaushik et al., 2021) | *skn-1* (Dodd et al., 2018; Kaushik et al., 2021) |
| *stdh-2* | 3.4 | 4.35E-08 | 4.78E-06 | *daf-2* (Senchuk et al., 2018), *daf-16* (Delaney et al., 2017; Kaplan et al., 2015) | *skn-1* (Dodd et al., 2018), *pmk-1* (Fletcher et al., 2019) |
| *T16G1.4* | 3.3 | 1.69E-11 | 1.08E-08 | *daf-2* (Gao et al., 2018; M. Seo et al., 2015; Sonoda et al., 2016),  *daf-16* [(Delaney et al., 2017; Kaplan et al., 2015; Kaushik et al., 2021; Li et al., 2019; Uno et al., 2013)](http://www.wormbase.org/species/species/expression_cluster/WBPaper00062193:daf-16(RNAi)_downregulated) | *skn-1* (Nhan et al., 2019) |
| *pgp-9* | 3.3 | 5.2E-15 | 1.11E-11 | *daf-2* (Sonoda et al., 2016), *daf-16* (Hibshman et al., 2017; Kaplan et al., 2015) |  |
| *pgp-7* | 3.1 | 2.48E-11 | 1.5E-08 | *daf-2* (Gao et al., 2018), *daf-16* (Kaplan et al., 2015) | *pmk-1* (Bond et al., 2014) |
| *lbp-8* | 3.1 | 5.67E-12 | 4.52E-09 | *daf-2* (Gao et al., 2018; Knutson et al., 2016; Sonoda et al., 2016), *daf-16* (Li et al., 2019) | *skn-1* (Dodd et al., 2018; Steinbaugh et al., 2015) |
| *R193.2* | 3.1 | 5.14E-10 | 1.82E-07 | *daf-2* (Knutson et al., 2016; Senchuk et al., 2018; Sonoda et al., 2016),  *daf-16* (S.-J. Lee et al., 2009, 2009; Li et al., 2019; Patel et al., 2008; Wu et al., 2016) | *skn-1* (Wu et al., 2016), *sek-1* (Wu et al., 2019) |
| *C32H11.9* | 3.0 | 2.17E-07 | 1.56E-05 | *daf-2* (Gao et al., 2018; Knutson et al., 2016; Son et al., 2018),  *daf-16* (Tepper et al., 2013) | *skn-1* (Dodd et al., 2018), *pmk-1*(Bond et al., 2014; Fletcher et al., 2019) |

Supplemental File 1c – Genes downregulated with a LFC <-3 in response to atRA in CITP N2

|  | | | | **Putative target of known regulatory pathway** | |
| --- | --- | --- | --- | --- | --- |
| **Gene** | **LFC** | **P-value** | **FDR** | **IIS (PI3K/Akt)** | **Nrf2 (p38 MAPK)** |
| *Y68A4A.13* | -5.8 | 3.78E-07 | 2.39E-05 | *daf-2* (Senchuk et al., 2018; M. Seo et al., 2015), *daf-16* (Kaplan et al., 2015; Kaushik et al., 2021; Li et al., 2019) | *skn-1* (Kaushik et al., 2021), *sek-1* (Wu et al., 2019) |
| *scl-20* | -4.6 | 5.32E-09 | 1.06E-06 | *daf-2* (Gao et al., 2018; Senchuk et al., 2018; M. Seo et al., 2015; Sonoda et al., 2016), *daf-16* (Amrit et al., 2016; Tepper et al., 2013) | *skn-1* (Nhan et al., 2019), *sek-1* (Wu et al., 2019) |
| *Y9C9A.16* | -4.1 | 2.62E-08 | 3.52E-06 | *daf-2* (Gao et al., 2018; Knutson et al., 2016; Senchuk et al., 2018; M. Seo et al., 2015; Son et al., 2018; Sonoda et al., 2016), *daf-16* (Delaney et al., 2017) | *sek-1* (Wu et al., 2019) |
| *col-183* | -4.0 | 1.13E-08 | 1.9E-06 | *daf-2* (Gao et al., 2018; Knutson et al., 2016; Senchuk et al., 2018; Son et al., 2018; Sonoda et al., 2016) | *skn-1* (Kaushik et al., 2021), *sek-1* (Wu et al., 2019) |
| *C54C8.12* | -4.0 | 6.67E-06 | 0.00021 | *daf-2* (Templeman et al., 2018) | *sek-1* (Wu et al., 2019) |
| *F54E4.2* | -3.9 | 6.05E-09 | 1.19E-06 | *daf-2* (Gao et al., 2018; Kaletsky et al., 2016; M. Seo et al., 2015) | *skn-1* (Nhan et al., 2019) |
| *dhs-26* | -3.9 | 9.61E-13 | 1.11E-09 | *daf-2* (Gao et al., 2018; Knutson et al., 2016; Senchuk et al., 2018; M. Seo et al., 2015; Son et al., 2018; Sonoda et al., 2016), *daf-16* (Kaushik et al., 2021) | *skn-1* (Nhan et al., 2019) |
| *F42A10.7* | -3.6 | 1.2E-08 | 1.92E-06 | *daf-2* (Golden and Melov, 2004; Knutson et al., 2016; Lan et al., 2019), | *skn-1* (Kaushik et al., 2021) |
| *pho-8* | -3.3 | 2.54E-07 | 1.78E-05 | *daf-2* (Knutson et al., 2016; Senchuk et al., 2018; Son et al., 2018), *daf-16* (Amrit et al., 2016; Tepper et al., 2013) | *skn-1* (Kaushik et al., 2021) |
| *C30G12.2* | -3.2 | 3.73E-12 | 3.4E-09 | *daf-2* (Gao et al., 2018; Knutson et al., 2016; Senchuk et al., 2018; M. Seo et al., 2015), *daf-16* (Hemphill et al., 2022) | *skn-1* (Kaushik et al., 2021), *sek-1* (Wu et al., 2019) |
| *irg-4* | -3.2 | 3.65E-13 | 4.65E-10 | *daf-2* (Gao et al., 2018; Knutson et al., 2016; Senchuk et al., 2018; M. Seo et al., 2015; Son et al., 2018; Templeman et al., 2018), *daf-16* (Kaushik et al., 2021; S.-J. Lee et al., 2009; Patel et al., 2008) | *skn-1* (Dodd et al., 2018; Kaushik et al., 2021), *pmk-1* (Bond et al., 2014; McEwan et al., 2016; Troemel et al., 2006) |
| *ftn-1* | -3.2 | 6.58E-11 | 3.35E-08 | *daf-2* (Knutson et al., 2016; Senchuk et al., 2018; M. Seo et al., 2015; Sonoda et al., 2016), *daf-16* (Kaplan et al., 2015) | *skn-1* (Kaushik et al., 2021; Nhan et al., 2019), *sek-1* (Wu et al., 2019) |

Supplemental File 1 – WaRGs response in *hsf-1(sy441)* or *aak-2* mutant backgrounds

|  | ***hsf-1*** | Maintained | Enhanced | Weakened | Lost | Flipped |
| --- | --- | --- | --- | --- | --- | --- |
| ***aak-2*** |  | 38.0% | 0.2% | 12.6% | 48.5% | 0.7% |
| Maintained | 77.9% | 208 | 1 | 68 | 197 | 1 |
| Enhanced | 3.6% | 4 | 0 | 6 | 12 | 0 |
| Weakened | 1.8% | 9 | 0 | 0 | 2 | 0 |
| Lost | 16.1% | 9 | 0 | 2 | 85 | 2 |
| Flipped | 0.7% | 2 | 0 | 1 | 0 | 1 |
